# Differential extracellular vesicle concentration and their biomarker expression of integrin α_v_/β_5_, EpCAM, and glypican-1 in pancreatic cancer models

**DOI:** 10.1038/s41598-024-65209-8

**Published:** 2024-06-20

**Authors:** Reed Jacobson, Sangdeuk Ha, Sakurako Tani, Shrinwanti Ghosh, Yagna P. R. Jarajapu, Randall E. Brand, Jiha Kim, Yongki Choi

**Affiliations:** 1 Departments of Physics, North Dakota State University, Fargo, ND 58108 USA; 2 Biological Sciences, North Dakota State University, Fargo, ND 58108 USA; 3 Pharmaceutical Sciences, North Dakota State University, Fargo, ND 58108 USA; 4 Molecular and Cellular Biology Program, North Dakota State University, Fargo, ND 58108 USA; 5 Department of Internal Medicine, University of Pittsburgh Medical Center, Pittsburgh, PA 15232 USA

**Keywords:** Biosensors, Biomarkers, Tumour biomarkers, Cell biology

## Abstract

Tumor-derived extracellular vesicles (EVs) show great potential as biomarkers for several diseases, including pancreatic cancer, due to their roles in cancer development and progression. However, the challenge of utilizing EVs as biomarkers lies in their inherent heterogeneity in terms of size and concentration, making accurate quantification difficult, which is highly dependent on the isolation and quantification methods used. In our study, we compared three EV isolation techniques and two EV quantification methods. We observed variations in EV concentration, with approximately 1.5-fold differences depending on the quantification method used. Interestingly, all EV isolation techniques consistently yielded similar EV quantities, overall size distribution, and modal sizes. In contrast, we found a notable increase in total EV amounts in samples from pancreatic cancer cell lines, mouse models, and patient plasma, compared to non-cancerous conditions. Moreover, individual tumor-derived EVs exhibited at least a 3-fold increase in several EV biomarkers. Our data, obtained from EVs isolated using various techniques and quantified through different methods, as well as originating from various pancreatic cancer models, suggests that EV profiling holds promise for the identification of unique and cancer-specific biomarkers in pancreatic cancer.

## Introduction

Cell-released lipid-bilayer extracellular vesicles (EVs) contain diverse molecules such as lipids, proteins, and nucleic acids, including endosome-origin small EVs (< 200 nm) known as exosomes^1–6^. EVs are believed to provide a means of intercellular communication and the transfer of macromolecules between cells, as well as implicated as contributing factors in the development of several diseases including cancer^3^. During the process of intercellular transmission, EVs either fuse with the plasma membranes of target cells or engage in interactions between surface proteins of EVs and receptors on recipient cells to deliver their contents including mRNA, miRNA, and DNA^3,7,8^, and initiate intercellular signaling^3,9^. As a result, the surface proteins of EVs and their contents have been considered critical factors in the identification of ‘disease-specific’ EV signatures.

Extensive research efforts have been dedicated to uncovering unique EV biomarkers. For example, several membrane-bound proteins, such as macrophage migration inhibition factor^10,11^, glypican-1 (GPC-1)^12,13^, tetraspanins^14,15^, and epithelial cell adhesion molecules (EpCAM)^14,16^, have been found to exhibit overexpression in circulating EVs associated with various types of cancer. Furthermore, recent proteomic analyses have identified distinct integrin (ITG) expression patterns on EVs related to tumor development and tropism for metastasis^17^. Remarkably, EVs isolated from the serum of pancreatic and breast cancer patients have shown elevated levels of ITGα_v_ (α_v_β_3_, α_v_β_5_) and ITGα_6_ (α_6_β_1_, α_6_β_4_), respectively^17^. These tumor-derived EVs serve critical roles in promoting cancer growth and facilitating metastasis by creating premetastatic niches that enhance organ-specific metastasis and redirect the distribution of metastatic cells^7,12,18,19^. Moreover, it has been shown that EV levels in serum increase early in the pancreatic intraepithelial neoplasia stage, preceding the formation of primary tumors that can be identified using current imaging techniques^7,12,18,19^. Consequently, tumor-derived EVs have emerged as promising blood-based biomarkers of liquid biopsy approaches aimed at screening and diagnosing pancreatic cancer, which is generally considered to be either incurable or difficult to detect in its potentially treatable early stages due to the absence of distinctive clinical symptoms and the lack of established screening and detection methods^20–22^. A recent study has demonstrated the potential of liquid biopsies for tracking glioma progression through EV phenotyping with surface plasmon resonance and atomic force microscopy techniques^23^.

While EV-based liquid biopsies hold great promise for advancing our understanding of cancer molecular biology, as well as for cancer diagnosis and prognosis, certain technical challenges persist in the separation, purification, and identification of EV-associated biomarkers from whole blood. These challenges arise due to the lack of standardized methods for EV isolation and characterization^3,24^. Additionally, issues such as low yield, poor quality, and preparation variability further complicate the process^25–27^. Moreover, the identification of tumor-specific EV biomarkers^12,28^ capable of distinguishing cancer from non-cancerous conditions remains elusive.

Currently, there are no clinically established EV biomarkers for the detection of pancreatic cancer. Here, we employed conventional EV isolation and quantification techniques to assess the size and concentration of EVs isolated from various sources, including pancreatic cancer cell lines, mouse models, pancreatic neuroendocrine tumors (PNET), and pancreatic ductal adenocarcinoma (PDAC) patients. Our study demonstrates that the quantification of EVs and the comparison of EV biomarker expression profiles of cancer and non-cancerous samples could improve the detection, identification, and molecular characterization of PDAC.

## Results

### EV concentration differed based on isolation methods

**Figure 1:**
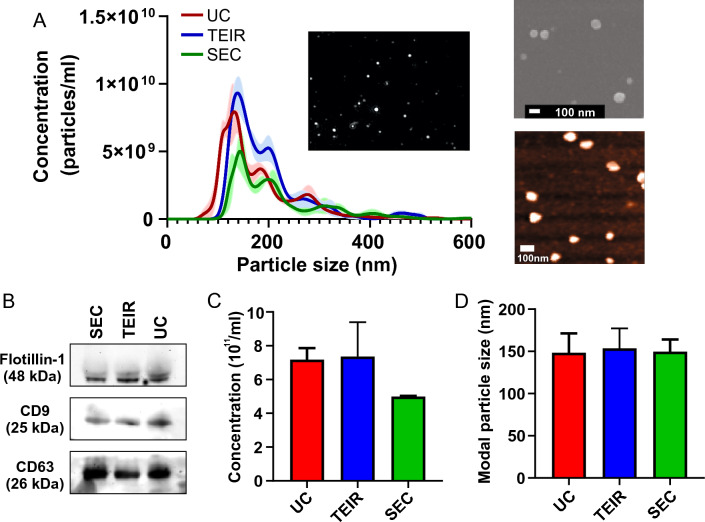
Comparison of three EV isolation methods on human plasma. (**A**) The size and concentration distribution of EVs isolated using UC, TEIR, and SEC methods, as determined by the NTA technique. The color shade represents the standard deviation (*n* = 3 technical replicates). The inset shows the light scattering image of the EVs. The top and bottom panels show SEM and AFM images of the EVs, respectively. (**B**) Immunoblot of CD9, CD63, and flotillin-1 EV marker proteins. 50 μg of EV protein extract was loaded per lane. (**C**) EV concentration of the identical PDAC patient plasma (*n* = 3 technical replicates), and (**D**) their modal EV size. Data are presented as mean ± standard deviation.

Three common EV isolation methods, ultra-centrifugation (UC), total exosome isolation reagent kit (TEIR), and size-exclusion chromatography (SEC), were evaluated using an identical plasma sample of PDAC patients. The EV size and concentration distributions were determined through nanoparticle tracking analysis (NTA) measurements (Fig. 1A). The overall profiles of the particle size and concentration distributions in samples isolated by each method displayed substantial overlap with each other. EVs larger than 500 nm were measured to be less than 1% of the total EV concentration for all measurements. Immunoblot analysis performed on the three EV samples revealed the presence of the EV marker proteins CD9, CD63, and flotillin-1 (Fig. 1B, Supplementary Fig. 1)^29^. All samples exhibited robust and consistent expression of flotillin-1 and CD63, with slight variations observed in the levels of CD9. This data validates the quality of EVs extracted via different methods. Notably, the total EV concentration varied depending on the isolation methods. Both UC and TEIR-based methods yielded approximately 1.5 times more EVs compared to the SEC-based method (Fig. 1C). Nevertheless, the peak value of the size distribution, representing the modal size of EVs, remained consistent across all three isolation techniques (Fig. 1A,D). Additionally, scanning electron microscopy and atomic force microscopy images (Fig. 1A) confirmed the consistent modal size of the EV observed in the NTA observation.

### Quantification methods influence EV concentration

**Figure 2:**
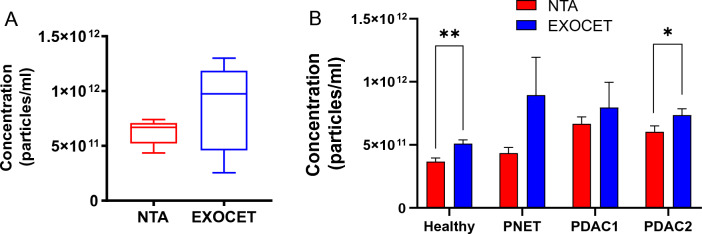
Comparison of two EV quantification methods. (**A**) The EV concentration of the identical PDAC patient, as determined by NTA and EXOCET techniques (*n* = 5 technical replicates). The statistics of the concentration between the two methods showed no statistical difference (not significant). (**B**) EV concentration of four different EV samples as determined by the two quantification techniques (Student’s *t*-test, *P < 0.05, **P < 0.01; *n* = 3 technical replicates per group). Data are presented as mean ± standard deviation. The statistics of the concentration between the two methods for PNET and PDAC1 showed no statistical difference (not significant) unless otherwise noted.

The quantification of EV size and concentration is commonly performed using the NTA method in various research fields related to EVs. However, an alternative EV quantitation method is the colorimetric enzyme assaying-based technique (EXOCET), which offers the advantage of assessing EV concentration using standard laboratory equipment, instead of a stand-alone NTA instrument. In this study, we examined both NTA and EXOCET to quantify EV concentrations isolated from the plasma of healthy, PNET, and PDAC patients using the UC method. When compared to NTA measurements, the EXOCET method provided EV counts that were up to three as high in all tested plasma samples (Fig. 2). Additionally, the EXOCET measurements exhibited a higher degree of variability in repeated measurements for the same EV samples compared to the NTA method (Fig. 2A). Nevertheless, there were no statistical differences observed in the quantification results between the two methods. More importantly, the overall trend in total counts across the four different EV samples remained consistent between both measurement techniques (Fig. 2B).

### Elevated EV concentration in pancreatic cancer with unchanged modal size

**Figure 3:**
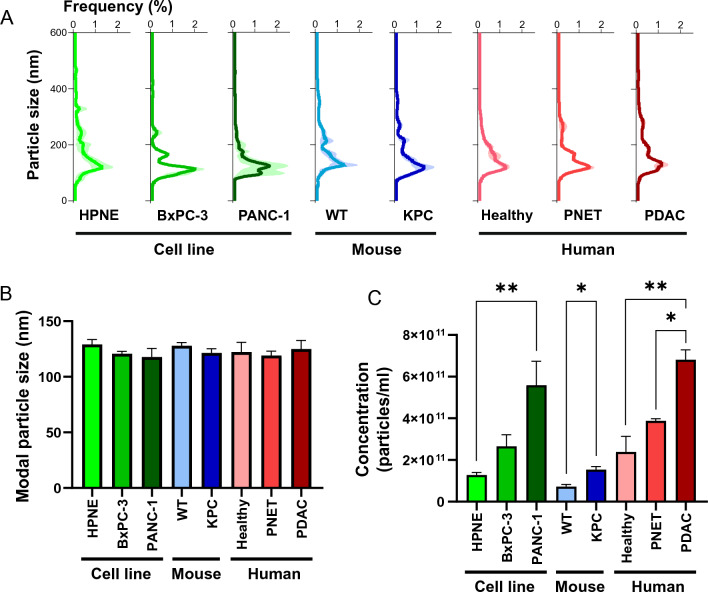
EV size and concentration in PDAC cell lines, mouse models, and patients. (**A**) The normalized size distribution of EVs isolated from each source, as determined by the NTA technique. The color shade represents the standard deviation (*n* = 3 per group). (**B**) The modal size and (**C**) concentration for each source. The statistical analysis was performed using either Student’s *t*-test or one-way ANOVA followed by Tukey’s post-hoc test (*P < 0.05, **P < 0.01; *n* = 3 per group). Data are presented as mean ± standard deviation. The statistics of the modal particle size among all EVs showed no statistical difference (not significant).

To compare the size and concentration of EVs between pancreatic cancer and non-cancerous conditions, we isolated EVs using the UC method from various sources including cell lines (HPNE, BxPC-3, PANC-1), mouse models (wild-type and KPC), and human plasma (healthy, PNET, and PDAC) and charactered them using the NTA method. Figure 3A depicts the normalized size distribution of each EV sample and compares the size probabilities between PDAC and non-cancerous EVs. Statistical analysis revealed no significant differences in size distribution across all EV samples. Moreover, a consistent modal size of approximately 125 nm was observed (Fig. 3B), regardless of the EV source.

Unlike the uniformity in the EV modal size, a notable contrast was observed in the overall EV concentration when comparing pancreatic cancer and non-cancerous EV samples (Fig. 3C). Specifically, the EV secretion by PANC-1 cells significantly exceeded that by HPNE cells. While the comparison of EV quantities between BxPC-3 and HPNE cells did not reach statistical significance (*P* = 0.11), the mean total EV count in BxPC-3 was consistently at least double that of HPNE cells. This pattern of elevated total EV quantities in pancreatic cancer was also observed in the mouse model (KPC vs. wild-type) and patient plasma (PDAC vs. healthy control) samples. Remarkably, the total number of EVs isolated from PDAC patient plasma was statistically distinguishable from both healthy and PNET cases.

### Unique EV biomarker expression profiles in pancreatic cancer

**Figure 4:**
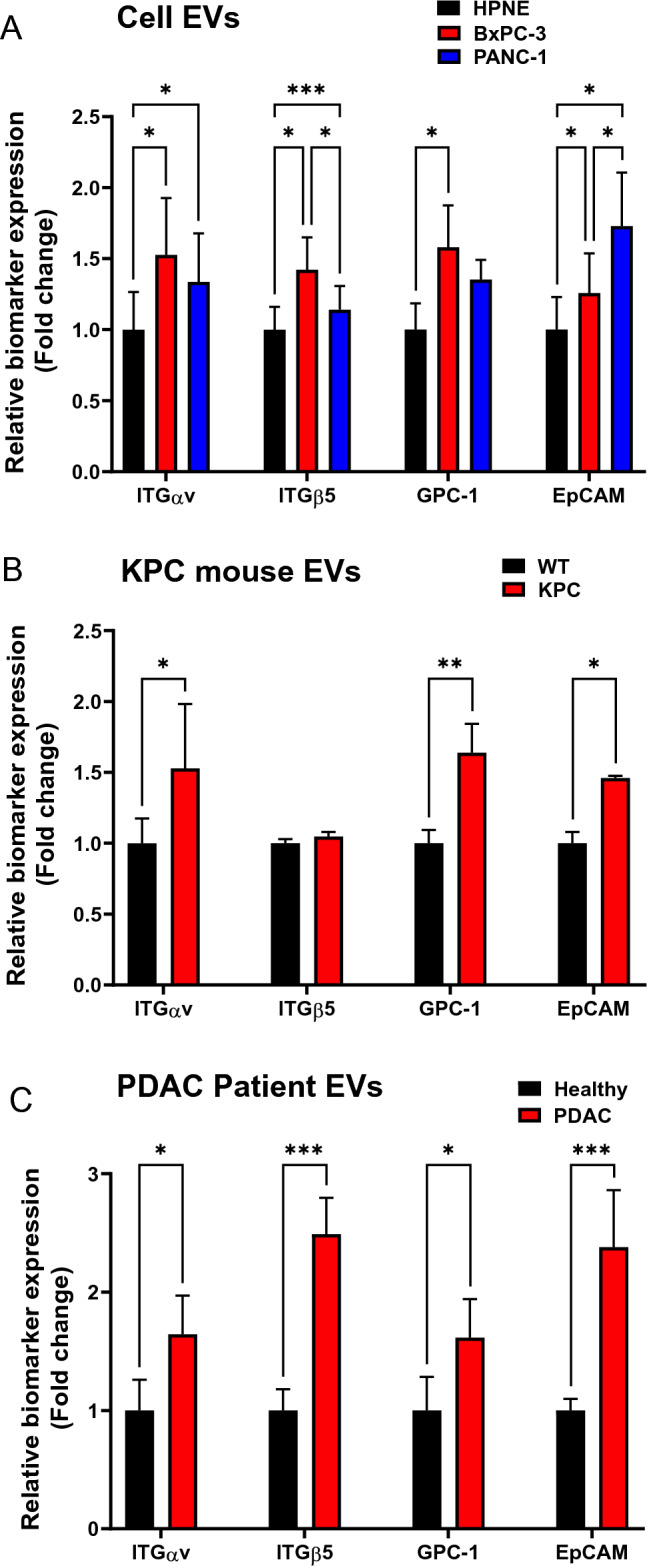
EV biomarker expressions in PDAC. Relative expression levels of ITGα_v_, ITGβ_5_, GPC-1, and EpCAM in EV protein lysates derived from (**A**) Cell lines (HPNE, BxPC-3, and PANC-1), (**B**) Mouse models (wild-type and KPC), and (**C**) Human plasmas (healthy and PDAC). Expression levels of each sample were normalized for EV marker flotillin level. The statistical analysis was performed using either Student’s *t*-test or one-way ANOVA followed by Tukey’s post-hoc test (*P < 0.05, **P < 0.01, ***P < 0.001; *n* = 3 per each group). Data are presented as mean ± standard deviation. The statistics of the GPC-1 expression level between HPNE and PANC-1, as well as the ITGβ_5_ expression level between wild-type and KPC mouse models showed no statistical difference (not significant) unless otherwise noted.

To assess the potential of EVs as biomarkers of pancreatic cancer, we investigated the relative protein expression levels of four EV biomarkers (ITGα_v_, ITGβ_5_, GPC-1, and EpCAM) in EVs isolated from PDAC cell lines, mouse models, and patient plasma (Fig. 4). Western blot analysis revealed significantly higher expression levels of all four biomarkers in EVs from the PDAC cell lines (BxPC-3 and PANC-1) compared to the normal HPNE cell line (Fig. 4A). Specifically, ITGα_v_, ITGβ_5_, GPC-1, and EpCAM exhibited statistically distinguishable expression differences between the BxPC-3 and HPNE cell lines. In the comparison between the PANC-1 and HPNE cell lines, ITGα_v_, ITGβ_5_, and EpCAM expression showed significant differences with a mild increase in GPC-1 expression observed in PANC-1 cells. Similarly, in mouse models, EVs from the KPC mouse model displayed significantly higher expression levels of ITGα_v_, GPC-1, and EpCAM compared to those from the wild-type mouse model (Fig. 4B, Supplementary Fig. 2). However, no significant difference was observed in the expression level of ITGβ_5_. Finally, we analyzed the protein expression of the four EV biomarkers in plasma EVs from healthy and PDAC patients (Fig. 4C, Supplementary Fig. 2). Notably, their relative expression significantly increased in PDAC EVs compared to healthy controls. This observation, consistent across cell lines, mouse models, and human samples, suggests their potential as diagnostic EV markers for PDAC.

## Discussion

We conducted a comprehensive evaluation of two quantification methods, NTA and EXOCET, and determined that both methods consistently provide reproducible and reliable results when analyzing samples of EVs from cancer and non-cancerous sources. Interestingly, our analysis revealed that the size and concentration distribution of EVs isolated through three different isolation techniques were also nearly identical, implying that neither the specific method of EV isolation nor the quantification technique significantly impacts the relative EV quantification or the size distribution.

Furthermore, our investigation showed a significant increase in the concentration of EVs isolated from PDAC cell lines, mouse models, and patient plasma when compared to their respective control groups. Markedly, the size distribution of these EVs remained consistent across all EV sources. These compelling observations suggest that pancreatic tumors may actively promote the secretion of EVs. Additionally, we observed significantly elevated expression levels of EV biomarkers ITGα_v_, ITGβ_5_, GPC-1, and EpCAM in EVs isolated from PDAC compared to non-cancerous conditions. This finding suggests a strong correlation between PDAC and the expression of these EV biomarkers.

Our results highlight the potential of both the relative EV concentration and the presence of these four EV protein signatures as specific biomarkers for the identification and prediction of PDAC. However, it is important to note that the total EV count and the quantity of EV biomarkers include contributions from both normal and tumor-derived EVs in PDAC patient plasma. Therefore, it is reasonable to assume that the differences in EV count and EV biomarker expression are predominantly driven by tumor-derived EVs. This enables us to assess the absolute increase in biomarker expression per tumor-derived EV in comparison to normal EVs.

By utilizing data from the fold-increase of the total EV count in PDAC (*N*_*c*_) and the fold-increase of relative biomarker expression levels in PDAC (*N*_*b*_), the fold-increase in biomarkers on individual tumor-derived EVs (*X*) compared to normal EVs can be estimated by *X* = (*N*_*c*_*N*_*b*_ − 1)/(*N*_*c*_ − 1), which represents the lower limit of the biomarker fold-increase in tumor-derived EVs. Given that both *N*_*c*_ and *N*_*b*_ for PDAC patients are approximately 2, we estimate that the fold-increase in biomarkers on individual tumor-derived EVs *X* is 3, indicating a minimum threefold increase in the biomarkers on these EVs.

In summary, EVs and their distinct biomarkers show promise for distinguishing PDAC from healthy conditions, highlighting their potential in both PDAC detection and advancing our understanding of PDAC biology.

## Materials and methods

### Cell culture

The PANC-1, BxPC-3, and hTERT-HPNE cell lines were obtained from ATCC and cultured as described previously^30^. All cells were cultured at 37 °C in a standard 5% CO_2_/95% air incubator. PANC-1 and BxPC-3 were cultured in high Glucose DMEM supplemented with 10% fetal bovine serum (FBS) and 1% penicillin–streptomycin. hTERT-HPNE was cultured in high Glucose DMEM supplemented with 10% FBS, 1% penicillin–streptomycin, and 0.75 μg/mL Puromycin.

### Animal studies

The disease progression and genotyping for the KPC mice were previously described^31^. KPC mice in C57BL/6J background were established as described previously at the NDSU animal facility^32^. WT C57BL/6J (000664) was purchased from Jackson Laboratory. The animals were kept in a pathogen-free, humidity (50–70%) and temperature (22–25 °C) controlled environment with a 12 h light/dark cycle. Fresh food and water will be provided ad libitum. KPC mice were monitored for tumor development using Vevo 3100 ultrasound. Blood samples were collected via retro-orbital puncture (100–400 ml, terminal bleeding), followed by euthanization for tumor collection. The average age of 3 KPC mice used the sample collection was four months. All animal experiments were reviewed and approved by the Institute of Animal Care and Use Committee at North Dakota State University. All experiments were conducted in accordance with ARRIVE guidelines. The conduct and reporting of the described experiments adhere to the “Guide for the Care and Use of Laboratory Animals” and the "U.S. Government Principles for the Utilization and Care of Vertebrate Animals Used in Testing, Research, and Training".

### Patient plasma

Plasma samples from patients with pathologically-verified pancreatic neuroendocrine tumors (PNET) and pancreatic ductal adenocarcinomas (PDAC), and healthy donors, who have no history of pancreatic disease or cancer, were collected at the University of Pittsburgh Medical Center as part of the Institutional Review Board approved Pancreatic Adenocarcinoma Gene Environment Risk (PAGER)—a prospective cohort study (IRB no. STUDY19070256). Written informed consent was obtained from patients to allow blood samples to be used for research. All experiments were performed in accordance with the IRB guidelines and regulations. The study was conducted in accordance with the Declaration of Helsinki, and the protocol was approved by the Ethics Committee of the University of Pittsburgh Medical Center.

### Ultracentrifuge EV isolation (UC)

Once cells were grown sub-confluent, cells were washed with PBS and the medium was changed to contain exosome-depleted FBS (Gibco™, A2720801) at 10%. The cells were then cultured for an additional 48 h. The culture supernatant was subjected to sequential centrifugation steps at 800*g* for 5 min and 2000*g* for 10 min, then filtered with 0.2 mm filters. The filtrate was subjected to ultracentrifugation at 28,000 RPM using an SW 28 Ti swinging bucket overnight to collect EVs. The supernatant was aspirated, and the pellet was resuspended in PBS. EVs collected from 30 ml of cell culture supernatant were resuspended in 200 µl of PBS. For both mouse and human plasma samples, 0.5 ml of plasma samples were centrifuged at 2,000 RPM at 4 °C for 30 min. The plasma sample was then mixed with PBS in a 1:20 ratio and filtered through a 0.2 μm filter. Filtrate was then centrifuged at 40,000 RRM overnight using Beckman SW 41 Ti. After ultracentrifugation, the supernatant was decanted, and the pellet was resuspended in 500 μl PBS. The purified EVs were then further analyzed.

### EV reagent isolation (TEIR)

A total exosome isolation reagent kit (4,478,360, Invitrogen) was used to isolate EVs according to manufacturer instructions. Briefly, 0.5 ml of plasma was centrifuged at 2000*g* for 30 min to remove all debris. Clarified plasma and provided reagent were mixed in a 5:1 ratio and incubated at 4 °C for 30 min. Precipitated EVs were then centrifuged for 10 min at 10,000*g*, and the supernatant was discarded. The EV pellet was then resuspended in PBS, and the purified EVs were further analyzed.

### Size exclusion chromatography EV isolation (SEC)

A qEV isolation column (qEV/35 nm, IZON Science) was used to isolate EVs according to manufacturer instructions. Briefly, 0.5 ml of plasma samples were centrifuged at 1500*g* for 10 min, then the supernatant was transferred to a new tube and centrifuged again at 10,000*g* for 10 min. Columns were equilibrated, clarified plasma was added to the column, and flow-through containing EVs was collected according to manufacturer instructions.

### Nanoparticle tracking analysis

The size and concentration distribution of EVs were determined by nanoparticle tracking analysis (NTA) using the NanoSight NS300 system (Malvern Panalytical Ltd, UK). The EV samples were diluted to 1000-fold in PBS for NTA measurements. The samples were infused with the syringe pump at a constant speed of 20 into the microfluidic flow cell equipped with a 532 nm laser and a high-sensitivity scientific CMOS camera. At least three videos per sample were recorded with a camera level of 11–13 for 30 s at 25 °C. All data were analyzed using NTA software (version 3.4) with a detection threshold of 4–6.

### EV colorimetric assay

An EXOCET exosome quantitation kit (EXOCET96A-1, SBI) was used to quantify the EV samples using the necessary reagents included in the kit according to manufacturer instructions. Briefly, isolated EVs were incubated in the provided lysis buffer to liberate associated proteins. Samples were then vortexed briefly and centrifuged at 1500*g* for 5 min. The supernatant was transferred to 96 well plates and incubated with reaction buffer for 15 min at room temperature. The reaction plate was read using xMark™ Microplate Absorbance Spectrophotometer immediately at 405 nm. Results were quantified by calculating the standard curve and plotting the sample readings on the standard curve.

### EV imaging

A 10–20 μL droplet of EVs in PBS was placed on a SiO_2_ substrate for 60 min at 4 °C. The samples were then washed with PBS and dried under nitrogen flow. Imaging of the samples was performed using atomic force microscopy (NTEGRA, NT-MDT)^33^ and scanning electron microscopy (JSM-7600F, JEOL).

### Western blot

EVs were lysed in NP40 cell lysis buffer (Invitrogen) containing 1 μM PMSF and 1 × Halt protease and phosphatase inhibitor cocktail (Thermo Fisher Scientific). Sample loading was normalized according to the Pierce BCA Protein Assay Kit (Thermo Fisher Scientific). Protein samples were separated using Mini-PROTEAN TGX Precast Gels (Bio-Rad) and transferred to nitrocellulose membrane using Bio-Rad Trans-Blot Turbo RTA transfer system (Bio-Rad). The protein blot was blocked using 5% non-fat dry milk in TBST for one hour at room temperature, then incubated overnight with the following primary antibodies: ITGα_v_ (ab179475, Abcam, 1:5000), ITGβ_5_ (ab184312, Abcam,1:1000), Anti-GPC-1 (PA528055, Thermo Scientific,1:2000), Anti-EpCAM (ab71916, Abcam,1:1000), Anti-CD9 (ab223052, Abcam, 1:2000), Anti-CD63 (ab216130, Abcam, 1:1000), and Anti-Flotillin-1 (ab133497, Abcam,1:1000). Blots were washed with TBST and then incubated with HRP-conjugated secondary antibody solution (1:3000) at room temperature for 1 h. After several washes with TBST, membranes were developed for 3–5 min using SuperSignal West Femto Maximum Sensitivity Substrate (Thermo Fisher Scientific). Images were then captured using the FlourChem FC2 imaging system (Alpha Innotech). Images were analyzed using ImageJ, and expressions of ITGα_v_, ITGβ_5_, GPC-1, and EpCAM were compared to Flotillin-1 and normalized.

### Statistical analysis

All data were expressed as mean ± standard deviation and analyzed using the Prism 8 (GraphPad software). The statistical significance was determined using Student’s *t*-test and analysis of variance (ANOVA) followed by a suitable post-hoc test. The p-values lower than 0.05 were considered statistically significant.

### Supplementary Information

Supplementary Figures.

## Data Availability

Data is available on request from the corresponding author.
