# Supplementary material for: Differential extracellular vesicle concentration and their biomarker expression of integrin αv/β5, EpCAM, and glypican-1 in pancreatic cancer models
Source: Sci Rep. 2024 Jun 20;14:14273. doi: 10.1038/s41598-024-65209-8 (PMC11189911; doi:10.1038/s41598-024-65209-8)
Supplement: Supplementary file 1 — Supplementary Figures. [file 41598_2024_65209_MOESM1_ESM.pdf]

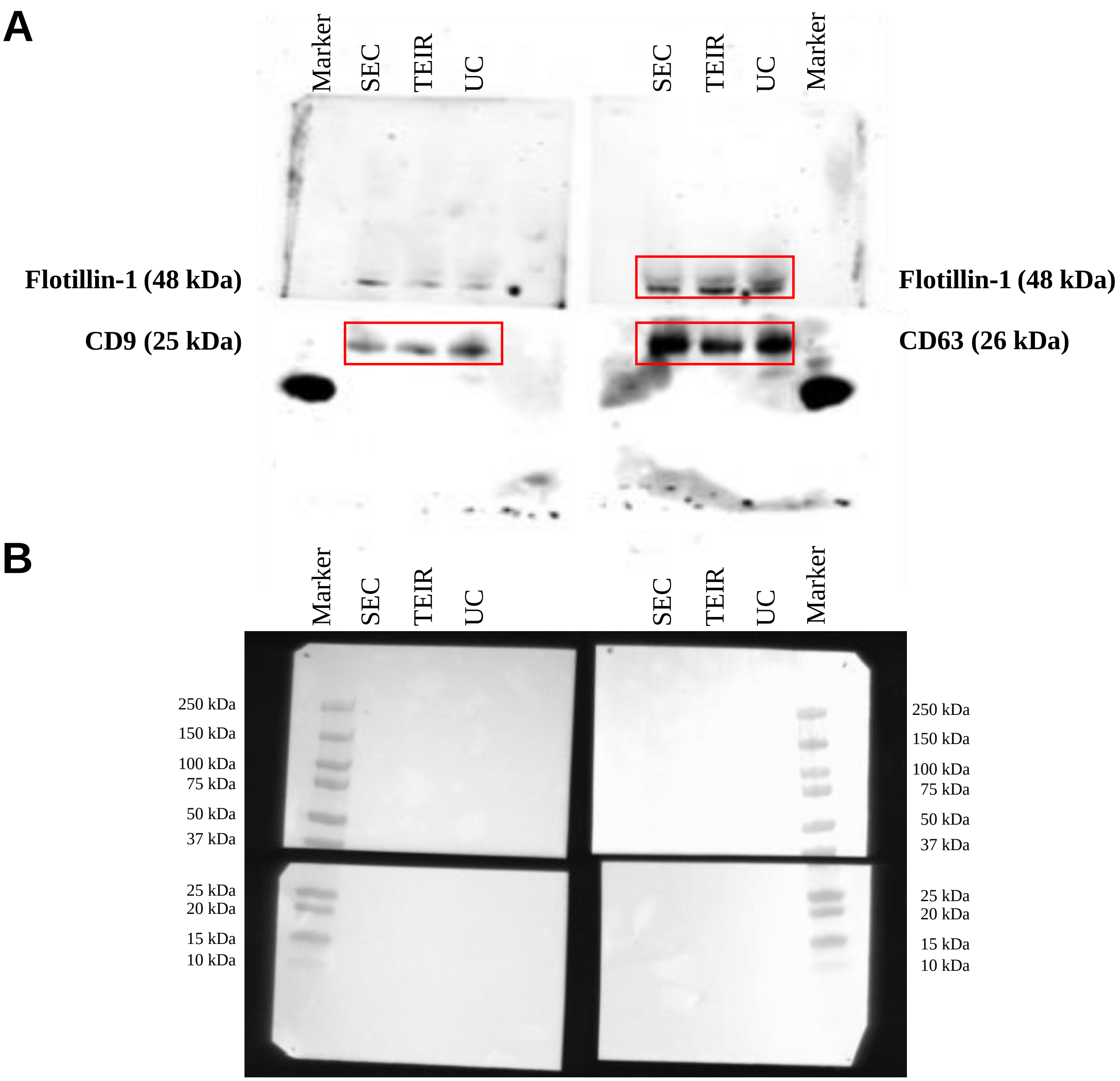

Supplementary Figure 1. Original Western blot images for **Figure 1B**. (**A)** Chemiluminescent images showing expression of exosome marker proteins, flotillin-1, CD9 and CD63, by exosomes isolated using indicated methods. The sample loading was duplicated, creating two sets of membranes due to almost identical sizes of CD9 (25 kDa) and CD63 (26 kDa). Red boxes indicate the area shown in Figure 1B. **(B)** Photographic images of the membranes shown in (A).

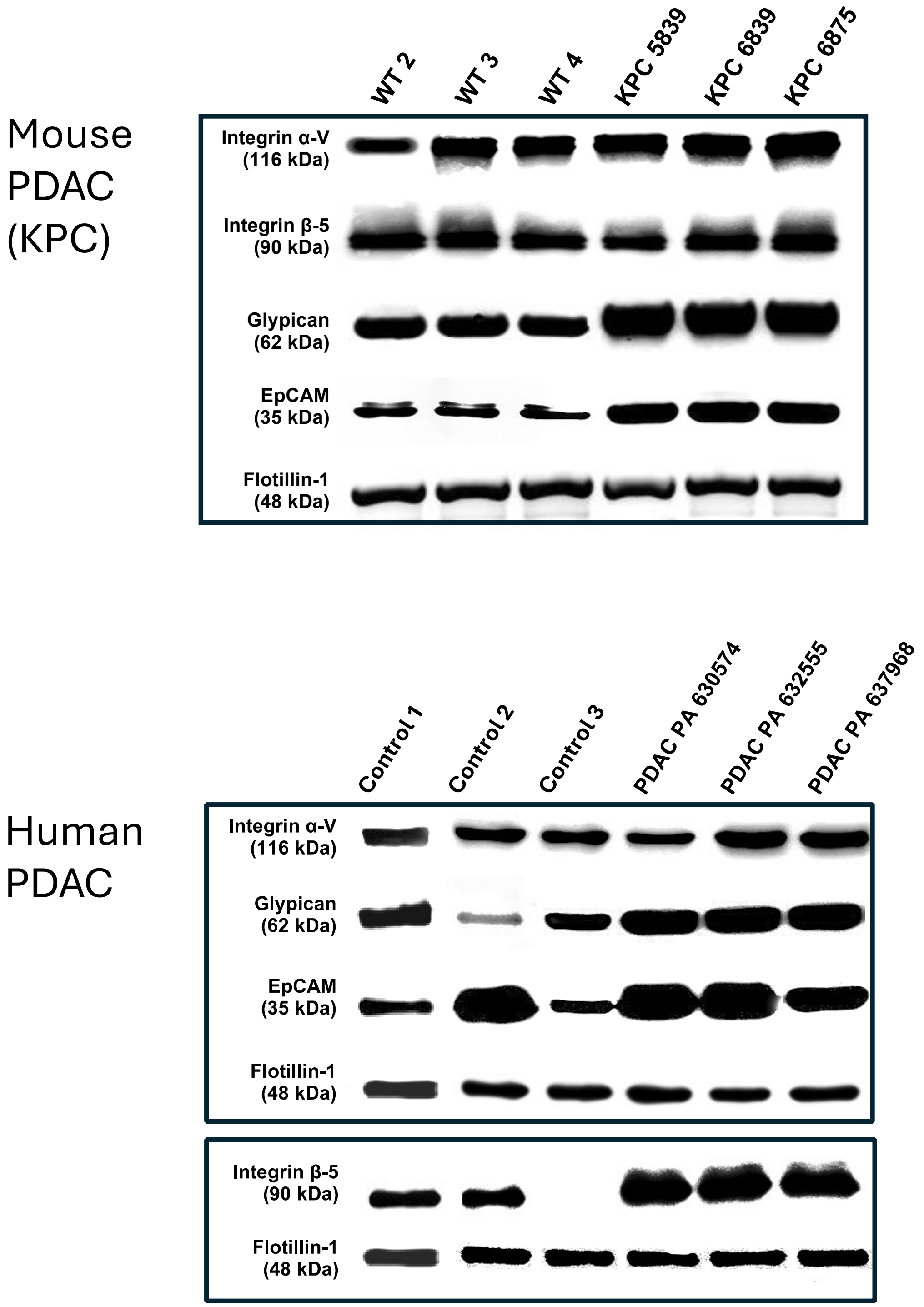

Supplementary Figure 2. Representative example of biomarkers expression from the PDACs.
